# A novel *Pinellia ternata* catalase gene *PtCAT2* regulates drought tolerance in *Arabidopsis* by modulating ROS balance

**DOI:** 10.3389/fpls.2023.1206798

**Published:** 2023-10-02

**Authors:** Juanjuan Xu, Ni Du, Tianci Dong, Han Zhang, Tao Xue, Fei Zhao, Fenglan Zhao, Yongbo Duan, Jianping Xue

**Affiliations:** ^1^ Anhui Provincial Engineering Laboratory for Efficient Utilization of Featured Resource Plants, College of Life Sciences, Huaibei Normal University, Huaibei, Anhui, China; ^2^ College of Agronomy & Resources and Environment, Tianjin Agricultural University, Tianjin, China

**Keywords:** *Arabidopsis thaliana*, catalase, drought, membrane integrity, *Pinellia ternata*, reactive oxygen species

## Abstract

Drought is one of the major abiotic stresses limiting agricultural production, particularly for shallow-rooted plants like *Pinellia ternata*. It damages plants via oxidative burst, but this effect could be mitigated by catalase (CAT). However, no studies have been reported on CAT homologs in *P. ternata*, a drought-sensitive plant species. In the present study, a novel CAT gene, *PtCAT2*, was functionally characterized via overexpression in *Arabidopsis* and analysis of cis-elements in its promoter. The isolated CAT gene was 1479 bp and encoded a protein containing 242 amino acids. The protein contains the CAT activity motif and the heme-binding site of a typical CAT, and the subcellular analysis indicated that the protein localizes at the cytoplasm and membrane. Moreover, the quantitative real-time reverse transcription PCR indicated that *PtCAT2* is expressed ubiquitously in *P. ternata* and is strongly induced by drought stress and abscisic acid (ABA) signals. *PtCAT2* overexpression enhanced the drought tolerance of *Arabidopsis*, as shown by the 30% increase in plant survival and a five-fold- increase in CAT activity. Moreover, *PtCAT2*-transgenic plants increased superoxide dismutase and peroxidase activities and reduced malondialdehyde, membrane leakage, and hydrogen peroxide (H_2_O_2_) (*P*<0.05). Furthermore, *PtCAT2*-transgenic plants showed higher tolerance to oxidative stress caused by exogenous H_2_O_2_ and retained higher chlorophyll and water contents than the WT. The mitochondria function was better maintained as presented by the higher oxygen consumption rate in transgenics under drought stress (*P*<0.05). The endogenous *CATs* and drought response-related genes were also upregulated in transgenic lines under drought stress, indicating that *PtCAT2* confers drought stress tolerance by enhancing the H_2_O_2_ scavenging ability of plants to maintain their membrane integrity. These results improve our understanding of the drought response mechanisms and provide a potential breeding strategy for *P. ternata* genetic improvement.

## 1 Introduction

Being sessile organisms, plants face various environmental stresses, including abiotic (such as drought, heat, salinity, and freezing) and biotic (such as fungi, bacteria, and viruses) factors, throughout their life cycles. Among these stresses, drought causes more annual loss in crop yield, making it the most limiting factor in agricultural production (Gupta et al., 2020; Yang and Qin, 2023). The shallow-rooted plants are sensitive to water deficit since their roots are distributed in the superficial soil layer.

*Pinellia ternata* is an Araceae plant species that has been broadly used in China, Japan, and Korea for thousands of years as a medicinal herb. Its tubers contain various bioactive compounds, including alkaloids, organic acids and lectins (Jin et al., 2012; Lee et al., 2016; Duan et al., 2019), conferring them with antitussive, antiemetic and anticancer activities (Lu et al., 2013). These multiple uses have ranked *P*. *ternata* among the top ten bulk Chinese medicinal materials (Zhang et al., 2021). Naturally, *P*. *ternata* grows in shaded environments. However, artificial cultivation has reduced the available shaded conditions for *P. ternata* growth. Thus, being a shallow-rooted plant, *P. ternata* suffers severe water deficit stress under artificial cultivation (Li et al., 2020; Zhou et al, 2020). Water deficit reduces tuber biomass, plant height, leaf area and propagation index (Chen et al., 2021), further decreasing productivity. Various plant growth regulators, including brassinolide (Guo et al., 2022), betaine (Zhang et al., 2014) and uniconazole (Cheng, 2010), have been applied to prevent the effects of water deficit in *P. ternata* production. However, the molecular mechanism underlying drought response in drought-sensitive plant species remains elusive.

Plants generate excessive reactive oxygen species (ROS), particularly the superoxide radical (O^2-^) and hydrogen peroxide (H_2_O_2_) (Kohli et al., 2019) under drought conditions, which, when not timely scavenged, accelerate the peroxidation of membrane lipids and even lead to cell death (Vijayaraghavareddy et al., 2022). Therefore, plants have evolved various ROS scavenging systems via enzymatic or non-enzymatic approaches (Mittler, 2017). Among the ROS, H_2_O_2_ is comparatively stable and diffuses freely in plants, severely threatening plant productivity under drought stress (Tian et al., 2016). Catalase (CAT, EC1.11.1.6), a tetrameric iron porphyrin protein present in all aerobically living organisms (Zaid and Wani, 2019), is an efficient ROS scavenger that directly catalyzes H_2_O_2_ degradation into O_2_ and H_2_O. This protects the plant cells against H_2_O_2_-caused membrane peroxidation. Therefore, modulating the CAT gene expression could be a promising approach for overcoming stress-caused H_2_O_2_ damage. Various studies have shown that plant CAT genes respond to various abiotic and biotic stresses (Yong et al., 2017; Sun et al., 2018); however, the responses are species-dependent. Moreover, the *CATs* responding to drought stress have not been fully investigated, particularly in drought-sensitive plants.

In this study, we functionally characterized a novel CAT gene, *PtCAT2*, through expression pattern analysis, subcellular location and overexpression analysis in *Arabidopsis*. The results will help understand the response mechanisms of *P. ternata* under water deficit conditions and developing drought-resistant germplasms.

## 2 Materials and methods

### 2.1 Plant materials and growth conditions

*P. ternata* (Thunb) Breit tubers were collected from the Experimental Farm of Huaibei University (E116.8, N34.0) and germinated in potted soil. The potted plants were kept at 25°C under a 16-h photoperiod and light intensity of 35 μmol m^-2^s^-1^. The leaves, roots, petioles, tubers and flowers were collected separately from the uniform-sized three-leaf stage seedlings treated with various abiotic stresses using previously published methods (Zhang et al., 2021). For temperature treatment, the potted plants were moved to RGC-1500C artificial climate incubators (Hefei Youke Instrument Equipment Co., Ltd, Hefei, China) at lower (4°C) and higher (40°C) temperatures, with those incubated at 25°C as control. The incubators had an illumination scheme of 16 h/8 h (light/dark) with a light intensity of 35 μmol m^-2^s^-1^. For other treatments, the three-leaf stage plants were rinsed in ddH_2_O and cultured in half-strength MS liquid medium containing 150 mM NaCl, 25% polyethylene glycol (PEG6000), or 100 μM ABA for salt, drought and abscisic acid (ABA) treatments, respectively, with those maintained in solely half-strength MS liquid medium as control. Whole plants were collected at 0, 6, 12, 24 and 72 h of treatment and rinsed in ddH_2_O. Three plants were bulked as one sample and three biological replicates were contained for each treatment. The sampled tissues or whole plants were frozen immediately in liquid nitrogen and preserved at –80°C for further analyses.

*Arabidopsis* Col-0 plants were grown in 2-L pots and cultured in RGC-1500C artificial climate incubators (Hefei Youke Instrument Equipment Co., Ltd, Hefei, China) at 22°C under a 16-h photoperiod and light intensity of 50 μmol m^-2^s^-1^.

### 2.2 Cloning of the PtCAT2 gene

A putative cDNA sequence of the *PtCAT2* gene was obtained from our previously published full-length transcriptome data (Xue et al., 2019) and amplified using the primer pair PtCAT2-Full-F/PtCAT2-Full-R (**Table S1**). The PCR products were purified and cloned into pEASY-T1 Simple Cloning Vector (Transgen, Beijing, China). The *PtCAT2* gene was also subjected to RT-PCR analysis at 94°C for 2 min, followed by 35 cycles of 94°C for 30 s, 58°C for 30 s, 72°C for 2 min, and a final extension at 72°C for 5 min.

### 2.3 Bioinformatics analysis of the PtCAT2 gene and its promoter

The open reading frame (ORF) of the *PtCAT2 gene* was obtained using the ORFfinder program (https://www.ncbi.nlm.nih.gov/orffinder/) and used to analyze the putative PtCAT2 protein sequence. The isoelectric point (pI), molecular weight (MW) and the grand average of hydropathicity (GRAVY) of the protein were then determined using the ExPASy ProtParam program (http://web.expasy.org/protparam/). Thereafter, the NCBI Conserved Domain (https://www.ncbi.nlm.nih.gov/Structure/cdd/wrpsb.cgi) and InterPro (http://www.ebi.ac.uk/interpro/) databases and the Motif Scan tool (http://myhits.isb-sib.ch/cgi-bin/motif_scan) were used to detect the catalytic active and heme-binding sites of PtCAT2. The subcellular localization of PtCAT2 was determined using the ProtComp (http://www.softberry.com/), CELLO (http://cello.life.nctu.edu.tw/) and PlantmPLoc (http://www.csbio.sjtu.edu.cn/bioinf/plant-multi/). Furthermore, SWISS-MODEL (http://swissmodel.expasy.org) was used to simulate the three-dimensional (3D) structure of PtCAT2. Functionally characterized CAT proteins (14) were used to construct the phylogenetic tree with the MEGA 7.0 program via the neighbor-joining method with 1000 bootstrap replicates.

The promoter sequence of *PtCAT2* was obtained from our genomic data (unpublished data), and a 2 kb sequence upstream of its transcription initiation site (ATG) was analyzed using the PlantCARE program (http://bioinformatics.psb.ugent.be/webtools/plantcare/html/) to predict the promoter cis-elements.

### 2.4 Subcellular localization assay

The ORF fragment of PtCAT2 was amplified from the pEASY-T1-PtCAT2 vector using primers PtCAT2-*Eco*RI-F/PtCAT2-*Sma*I-R (**Table S1**). The amplified product was then transformed into the multiple cloning sites upstream of the green fluorescent protein (GFP) of the pCAMBIA1302 vector via double enzyme digestion and ligation. Thereafter, the obtained pCAMBIA1302-PtCAT2-GFP construct was verified by enzyme digestion, and the expression vector was transformed into onion cells according to a previously published method (Xu et al., 2014). The GFP fluorescence of the onion cells was then observed under a confocal microscope (FV1000SP, Olympus, Japan) 24 h later.

### 2.5 RNA isolation, cDNA synthesis and quantitative reverse transcription PCR analysis

RNA isolation and cDNA synthesis were performed according to our previously published method (Zhang et al., 2021). Briefly, RNA from *P. ternata* and *Arabidopsis* plants was extracted using the standard TRIzol method. The obtained RNA was used as the template for cDNA synthesis, conducted using the 5×All-In-One MasterMix transcription and AccuRT Genomic DNA Removal Kits (Cat#G492) (ABM, Richmond, BC, Canada), following the manufacturer’s instructions.

Quantitative real-time RT-PCR (qRT-PCR) was performed on an ABI7500 fast Real-Time PCR system (Applied Biosystems, Foster City, CA, USA) using a 2×AceQ qPCR SYBR Green Master Mix (Vazyme, Piscataway, NJ, USA). The ribosomal 18S gene (*Pt18S*) and β-tubulin (*AtTUB*) were used as internal controls for *P. ternata* and *Arabidopsis*, respectively, and the relative expression of the target genes was calculated using the 2^−ΔΔ^ *^CT^* method (Livak and Schmittgen, 2001). Three technical replicates and three biological replicates were used for each treatment. The primer sequences used for the qRT-PCR are listed in Supplementary (**Table S1**).

### 2.6 *Arabidopsis* transformation and selection of the homozygous lines

The *PtCAT2* cDNA, amplified from the pEASY-T1-PtCAT2 vector using the primer pair PtCAT2-Smal I-F/PtCAT2-Xba I-R, was purified and inserted into the multiple cloning sites of the pCAMBIA2300-35S-GUS-CaMVterm vector (Duan et al., 2015). The obtained recombinant vector and the empty vector pCAMBIA2300-35S-GUS-CaMVterm were verified by double enzyme digestion and then transformed into *Agrobacterium-*tumefaciens strain EHA105. Thereafter, *Arabidopsis* (Col-0) was transformed with the *Agrobacterium* cells containing the vectors via the floral-dip method (Clough and Bent, 1998). The transgenic plants were screened by plating the surface-disinfected seeds onto half-strength MS media containing 10 mg·L^-1^ kanamycin, and the positive plants were transplanted into pot soil for seed harvesting. The seeds obtained from individual plants were then screened on 10 mg·L^-1^ kanamycin until the T_3_ homozygous lines were obtained.

### 2.7 Drought tolerance analysis of the transgenic plants

The T_3_ transgenic seeds were surface-disinfected, vernalized and germinated on half-strength MS media containing 25% (m/v) polyethylene glycol (PEG)-6000. Their germination rates were recorded, and the images were captured two weeks after the treatments.

For the water deficit treatment, 2-week-old seedlings from T_3_ homozygous transgenic *Arabidopsis* lines and WT were transplanted into six-hole plastic plates filled with a mixture of soil and vermiculite (3:1). Five plates containing 30 plants were included for each treatment in triplicate. The seedlings were maintained at 22°C under 70% humidity and a 16 h/8 h light/dark photoperiod and subjected to water deficit treatment after one week. Leaves were harvested after seven days of drought stress, frozen in liquid nitrogen, and preserved at −80°C for further analysis. After ten days of drought stress, the survival rates of the seedlings were recorded, and the images were captured.

### 2.8 Determination of ROS, antioxidant enzyme activity and membrane integrity

The activities of antioxidant enzymes superoxide dismutase (SOD), peroxidase (POD), CAT and the content of malondialdehyde (MDA) were measured as described previously (Tian et al., 2022). Histochemical staining with diaminobezidin (DAB) and nitrotetrazolium blue chloride (NBT) was performed using the method by Zhang et al. (2021). H_2_O_2_ and O_2_^·-^ levels were analyzed by using the detection kits (Nanjing Jiancheng Bioengineering Institute, Jiangsu, China).

The electrolyte leakage (El) was measured following a previously published method (Zhang et al., 2021). Briefly, the leaves were sliced, immersed in deionized water, and incubated at room temperature on a shaker (20×*g*) for 1 h. The initial conductivities of the treatment (C1) and blank (CK1) samples were measured by a conductivity meter (DDSJ-318, INESA Scientific Instrument Co., Ltd., China) before boiling. Thereafter, the samples were boiled for 10 min and cooled down to room temperature, and their second conductivities (C2 and CK2) were recorded. The El was presented as the relative conductance (C) calculated using the following equation: C (%) = (C1 − CK1)/(C2 − CK2) × 100.

The water loss rate was measured by weighing the leaf samples at 0, 1, 2, 3, 4 and 5 h after their detachment from the stems, and the relative water loss rate was presented as the ratio of leaf weight at each time point relative to that at 0 h.

### 2.9 Mitochondria isolation and respiration assays

The mitochondria isolation and respiratory measurements were performed following a previously published method (Lyu et al., 2018). The leaves of two-week-old wild-type and transgenic *Arabidopsis* seedlings grown at 22°C were used to isolate the mitochondria. The respiration of the isolated mitochondria was determined using a Clark-type oxygen electrode (Hansatech, King’s Lynn, England). The reaction buffer contained 0.3 M mannitol, 10 mM TES-KOH (pH 7.5), 3 mM MgSO_4_, 10 mM NaCl, 5 mM KH_2_PO_4_ and 0.1% BSA. Succinate (SA) at 5 mM and NADH at 1 mM were the respiratory substrates. The buffer was added with 5 mM SA and 1 mM NADH, and then incubated at 25°C for reaction.

### 2.10 Measurement of chlorophyll content under oxidative stress

The exogenous H_2_O_2_-caused oxidative damage on chlorophyll was measured following the method by Tian et al. (2022). Briefly, whole leaves obtained from the same positions on 2-week-old transgenic and WT *Arabidopsis* plants were incubated in 10 mL solution containing 0, 0.5% and 1% H_2_O_2_ for 24 h at 25°C under a 16 h/8 h light/dark photoperiod. Thereafter, the images were captured, and the chlorophyll content of the leaves was determined, as described previously (Lichtenthaler, 1987).

### 2.11 Statistical analysis

Data were analyzed using the Statistical Analysis System software (IBM SPSS Statistics 21.0, IBM Corp., NY, USA), and the significant differences among treatments were determined using the Students’ t-test or Duncan’s multiple range test (*P* < 0.05). The data are presented as means ± SD values of three biological replicates (n=3).

## 3 Results

### 3.1 Bioinformatics analysis of the PtCAT2

From a *P. ternata* transcriptome data (Xue et al., 2019), 13 CAT homologs were identified. Of them, i1_HQ_Pts1_c90512/f3p3/1940 (annotated as *PtCAT2*), showed a remarkable drought-inducible expression profile (**Figure S1**). *PtCAT2* cDNA consisted of 1479 bp long ORF (**Figure S2**) encoding 492 amino acids. Its molecular weight and isoelectric points were 57 KDa and 6.91, respectively. Moreover, the protein had an aliphatic index of 37.65 and a GRAVY of –0.570, suggesting that PtCAT2 is a hydrophilic protein. The sequence alignment analysis identified the CAT activity motif (FARERIPERVVHARGAS) and heme-binding site (RIFAYADTQ) of PtCAT2 at positions 54–70 and 344–352, respectively, consistent with the other analyzed CATs (**Figure S3**). This implies that PtCAT2 is a typical CAT.

Furthermore, the SWISS-MODEL analysis showed that the three-dimensional (3D) structure of PtCAT2 has four typical subunits (**Figure S4**). The PtCAT2 aligned with the template protein 4qol.1.A from amino acids 11 to 490, covering 97.36% of the template. Moreover, the PtCAT2 protein shared a high homology (48.54%) with the template protein, and it had a GMQE value and QMEAN Z-Score of 0.81 and –1.08, respectively, confirming the high reliability of the predicted structure.

Phylogenetic analysis suggested that CATs from *Triticum monococcum*, *Triticum aestivum*, *Brassica oleracea*, *Saccharum hybrid*, *Zea mays*, *Oryza sativa*, *Setaria italica*, *Nelumbo nucifera*, *Selenicereus undatus*, *Arabidopsis thaliana*, *Hordeum vulgare*, *Sorghum bicolor* and *Aeluropus littoralis* could be divided into two classes, consistent with previous publications (Sun et al., 2018). PtCAT2 clustered with class II CATs, though it did not have a high similarity with other CATs (the highest homology was 82% homology with the *T. aestivum* TaCAT) (**Figure S5**), demonstrating that PtCAT2 is a hypothetical class II CAT.

### 3.2 PtCAT2 expresses ubiquitously in *P. ternata* and is highly induced by drought and ABA

**Figure 1:**
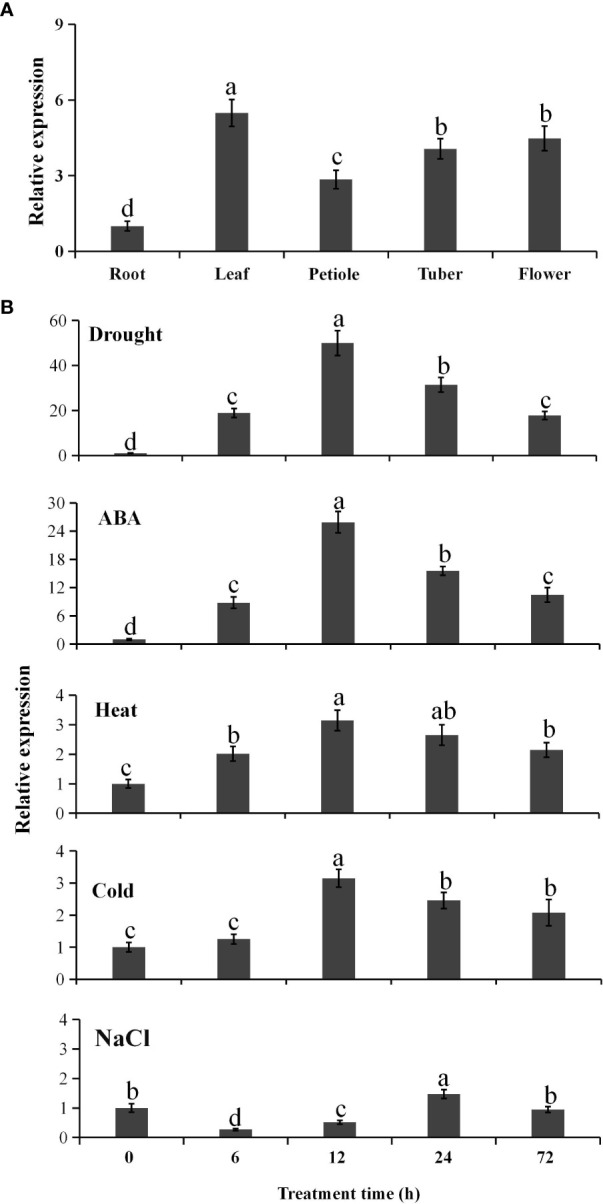
Spatial expression profile of *PtCAT2* and its inducible expression pattern under abiotic stresses. **(A)** The root, leaf, petiole, tuber, and flower from two-month-old *Pinellia ternata* plants (three-leaf stage) were collected for analysis. The relative expressions in each tissue were calculated using the 2^−ΔΔ^ *^CT^* method. **(B)** The 3-week-old seedlings were cultured in 1/2 MS liquid medium containing 150 mM NaCl or 25% polyethylene glycol (PEG6000) or 100 μM abscisic acid (ABA) for salt, drought, and ABA treatment respectively. The potted 3-week-old seedlings exposed to 4°C or 40°C for cold or heat stress respectively. Whole seedlings were collected at the indicated time points for RNA isolation. *Pt18SrRNA* was used as an internal control to normalize the relative expressions. Values are presented as means ± SD (n = 3). Different lower cases indicate significant differences at *P*<0.05.

Quantitative real-time RT-PCR indicated that *PtCAT2* was ubiquitously expressed in *P. ternata* (**Figure 1A**) and had a 5.5-fold higher expression in the leaves, followed by flowers (4.9-fold), tubers (4.1-fold) and petioles (2.8-fold), compared with that in roots.

To identify the abiotic stress response of *PtCAT2*, we exposed the *P. ternata* plants to various abiotic stresses and used qRT-PCR to analyze the relative expression level of *PtCAT2*. When stressed with 25% PEG, we observed that the *PtCAT2* mRNA level significantly increased within 72 h of the treatment, with the expression peak at 12 h (a 51-fold induction) (**Figure 1B**). *PtCAT2* was also remarkably induced by 100 μM ABA, with a 25-fold expression at 12 h. The *PtCAT2* mRNA abundance also increased to some extent when treated with salt (150 mM NaCl), low temperature (4°C) and heat (40°C); however, the inductions were less than 3.5-fold (**Figure 1B**). Thus, *PtCAT2* could be remarkably inducible by drought and ABA but not by the other abiotic stresses tested in this study.

To further analyze the drought-induced expression of *PtCAT2*, we used the PlantCARE software to predict the possible acting cis-elements in its promoter. We identified various cis-elements in the *PtCAT2* promoter (**Figure S6**), which regulate its response to abiotic stresses. Among these were three MYCCONSENSUSAT (CANNTG), two MYCATRD22 (CACATG) and one ABRE, which may be involved in drought and ABA response. There were also GT, ACGTATERD1 and GAATTC cis-elements, which may be associated with the weak response of *PtCAT2* to salt, cold and heat stresses. These results imply that the significant induction of *PtCAT2* under drought could be attributed to MYCCONSENSUSAT or MYCATRD22 and probably mediated by ABA.

### 3.3 Subcellular location of PtCAT2

**Figure 2:**
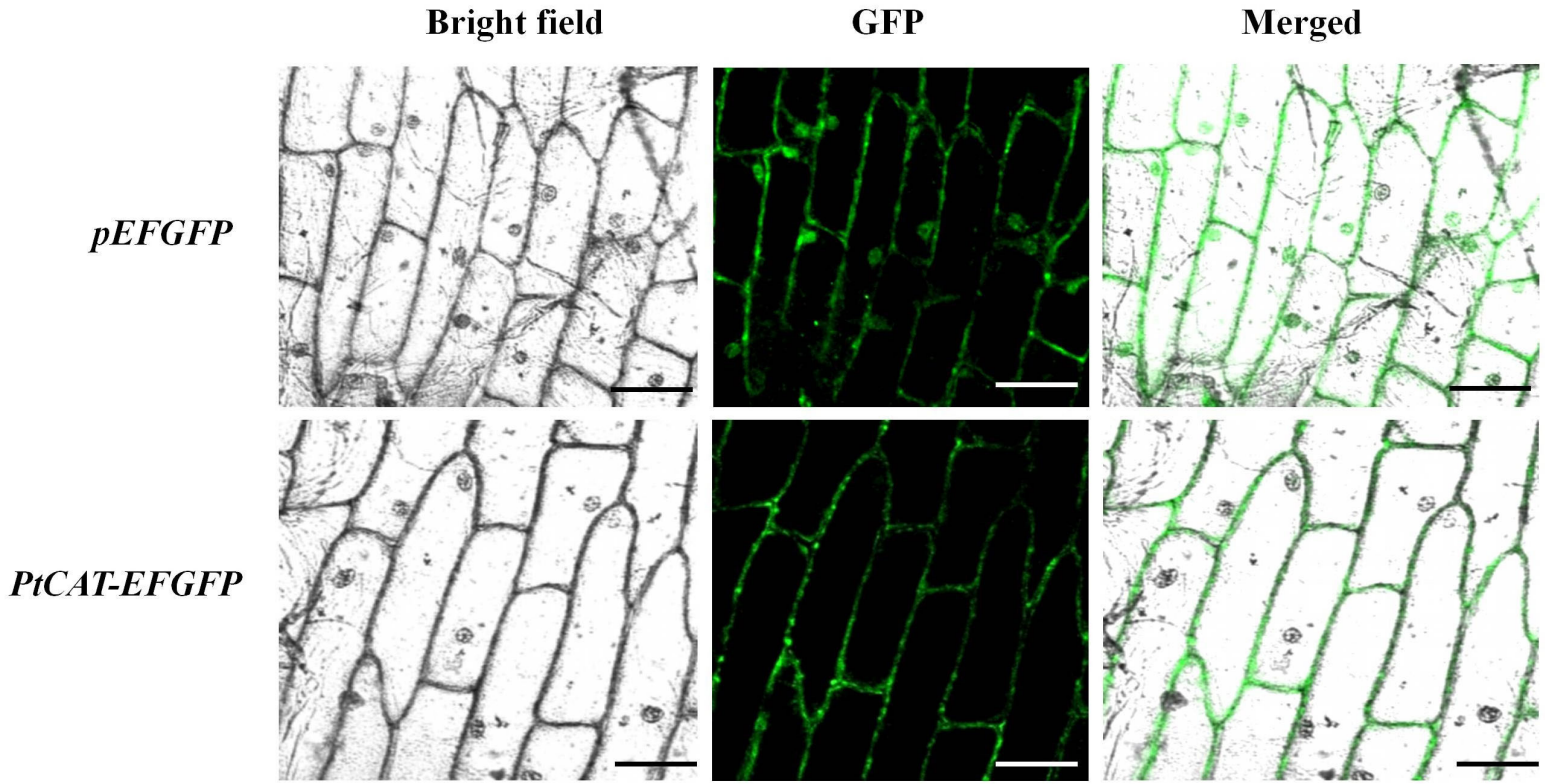
Subcellular localization of PtCAT2 and empty vector pEFGFP in onion after 12 h of infiltration. Images of epidermal cells were captured under visible, green fluorescence and merged light. Bar: 50 μm.

The plant expression vector harboring the fused *PtCAT2*-*GFP* cassete (**Figure S7**) was transformed into onion epidermal cells. The fluorescence results showed the presence of GFP signals in the cell membrane and cytoplasm, not in the nucleus (**Figure 2**). This is consistent with the results of the protein subcellular localization prediction tool (PSORT), which showed a 78.7% probability of localization in the cytoplasm and 4.3% of localization in the plasma membrane.

### 3.4 Overexpression of the PtCAT2 gene enhanced the drought tolerance of the transgenic *Arabidopsis*

**Figure 3:**
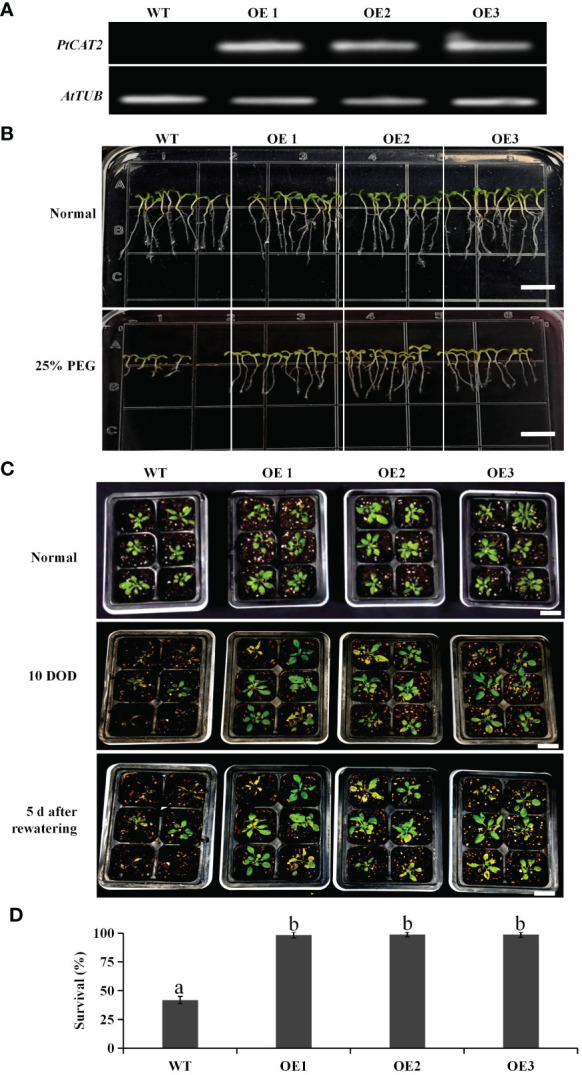
Identification of *PtCAT2-*transgenic *Arabidopsis* lines and drought tolerance analysis of wild-type (WT) and transgenic seedlings. **(A)** The relative expression of *PtCAT2* was assessed by RT-PCR using *Arabidopsis* β-tubulin (*AtTUB*) as an internal control. **(B)** Germination performance of WT and transgenics on 1/2MS medium with or without 25% PEG. The pictures were taken at 14 d after culture. Bar: 0.5 cm. **(C)** Three-week-old *Arabidopsis* seedlings were subjected to water deficit treatment for 10 d and a followed 5 d of rewatering culture. Bar: 3 cm. **(D)** Survival of *Arabidopsis* plants recorded after 5 d of rewatering following 10 d of water deficit. Data are the mean of three biological replicates. OE1, OE2 and OE3 represent three independent transgenic lines. Values are presented as means ± SD values (*n* = 3). Different letters indicate significant differences at *P* < 0.05.

The T_3_ homozygous lines of transgenic *Arabidopsis* were identified with RT-PCR, and three lines (OE1, OE2 and OE3) were selected for further analysis (**Figure 3A**). The seeds of the three lines were subjected to drought stress via germination on a half-strength MS medium containing 25% PEG. We found that the *PtCAT2*-transgenic and WT seeds had no significant difference during germination under normal conditions. However, the germination rate of the *PtCAT2*-transgenic *Arabidopsis* seeds was remarkably higher than that of the WT under PEG-simulated drought conditions (**Figure 3B**).

The drought tolerance of the transgenic *Arabidopsis* plants was further evaluated through water deficit treatment (**Figure 3C**). We found that most WT plants withered or even died after 10 days of water deficit treatment, while the transgenic plants maintained normal growth. Following a 5 d recovery after rewatering, over 95% of the transgenic plants recovered, compared to the 41% survival of the WT (**Figure 3D**). Thus, these results indicate that overexpressing *PtCAT2* significantly enhanced the drought tolerance of *Arabidopsis* plants.

### 3.5 PtCAT2 overexpression alleviated free radical damage on the membrane under drought stress

**Figure 4:**
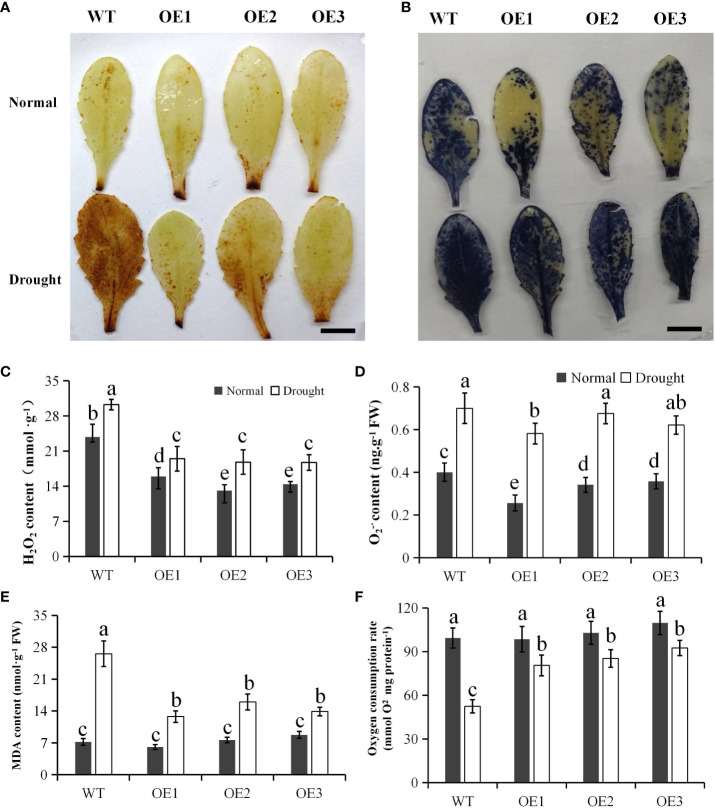
Measurements of ROS and oxygen consumption rate in WT and transgenic plants under heat stress. The leaves were sampled from 3-week-old plants grown under normal growth conditions and subjected to 7 d of water deficit stress. **(A)** DAB staining of intact leaves. Bar: 1 cm. **(B)** NBT staining of intact leaves. Bar: 1 cm. **(C)** H_2_O_2_ content. **(D)** O_2_^-·^ content. **(E)** MDA content. **(F)** Oxygen consumption rate of mitochondria.

Since the *Arabidopsis* plants overexpressing *PtCAT2* had enhanced drought tolerance, we subjected their leaves and those of the WT to DAB and NBT staining. In DAB staining, the transgenic leaves had lighter stains than the WT under drought stress but did not differ from the WT under normal conditions (**Figure 4A**). However, the NBT-stained leaves showed no much difference between transgenics and WT (**Figure 4B**). The H_2_O_2_ content was lower in transgenic leaves than in WT under normal and drought conditions (*P*<0.05) (**Figure 4C**), though O_2_^·-^ content did not show significant difference between transgenics and WT (**Figure 4D**) except OE1. The results further confirm that overexpressing *PtCAT2* efficiently degrades H_2_O_2_ in *Arabidopsis* under drought stress but not significantly affects O_2_^·-^ production.

Malonaldehyde (MDA) is an important indicator of the degree of lipid peroxidation, commonly used to estimate membrane damage under oxidative stress (Ashraf et al., 2017). Although there was no difference between the MDA content of the WT and transgenic plants under normal conditions, the transgenic plants had significantly lower MDA content than WT plants under drought stress (*P*< 0.05) (**Figure 4E**). Further, the oxygen consumption rate was investigated to test the effect of *PtCAT2* expression on the mitochondrial function. Three transgenic lines maintained higher oxygen consumption rate under drought stress in comparison with WT (*P*< 0.05) (**Figure 4F**). These data indicate that overexpression of *PtCAT2* alleviates the membrane damage and maintain the mitochondrial function under drought stress.

**Figure 5:**
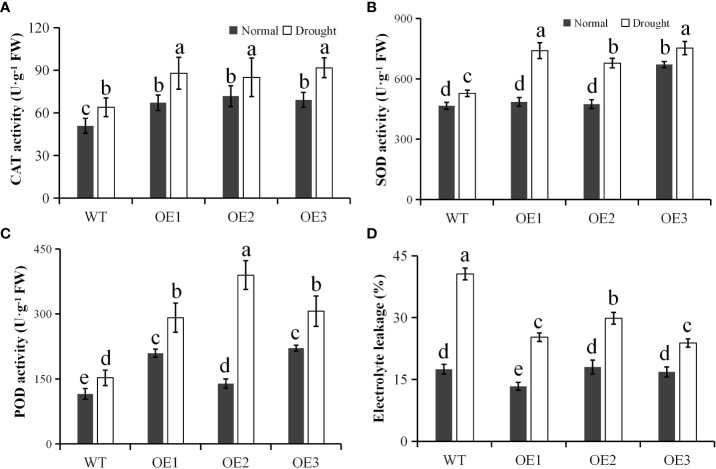
Antioxidant enzyme activities and membrane integrity. **(A)** CAT activity. **(B)** SOD activity. **(C)** POD activity. **(D)** Electrolyte leakage. OE1, OE2 and OE3 represent three independent transgenic lines. Values are presented as means ± SD values (*n* = 3). Different letters indicate significant differences at *P* < 0.05. Bar: 1 cm.

We also determined the CAT activity of the transgenic lines and WT, and found that similar to the mRNA level, the CAT activities of the three transgenic lines were significantly higher than WT under normal and drought stress conditions (**Figure 5A**). This showed the enzymatic correlation between PtCAT2 and drought tolerance. We also quantified the activities of SOD and POD. The result showed that the POD and SOD activities were similar between WT and transgenic plants (*P*>0.05) under normal conditions. However, when stressed with the water deficit treatment, the POD and SOD activities increased in WT or transgenic plants, but the increment was significantly higher in transgenic plants compared to WT plants (*P*<0.05) (**Figures 5B, C**). The El was measured to determine the membrane integrity of the plants under water deficit stress. We found no significant differences between the El values of the WT and transgenic leaves under normal conditions; however, the transgenic leaves presented significantly lower El values under drought treatment (*P*< 0.05) (**Figure 5D**). These data suggest that *PtCAT2* overexpression could alleviate the membrane damage by enhancing the free radical scavenging ability of *Arabidopsis*, thus improving its drought stress tolerance.

### 3.6 PtCAT2 overexpression enhanced the antioxidative and water retention capacities of Arabidopsis

**Figure 6:**
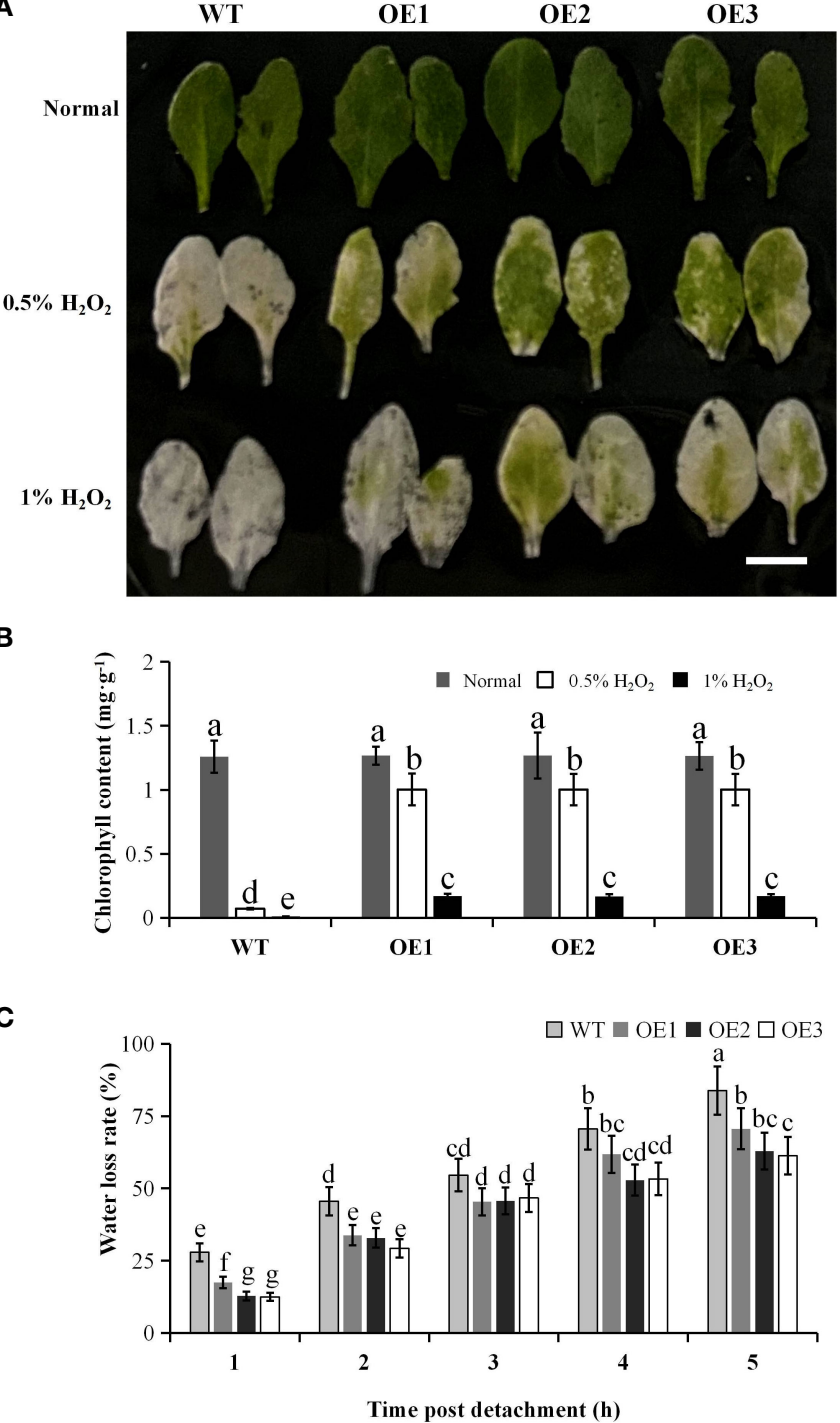
Oxidative stress tolerance experiment and water loss rate of 3-week-old transgenic lines and wild-type (WT) plants. **(A)** Intact leaves with petioles from 3-week-old plants were exposed to 0, 0.5%, and 1% H_2_O_2_ solutions for 24 (h) Bar: 1 cm. **(B)** Chlorophyll content in leaves was determined at 24 h after treatment. **(C)** The water loss rate of the leaves was measured by recording the weights of leaves at the indicated time points after they detached. OE1, OE2 and OE3 represent three independent transgenic lines. Values are presented as means ± SD values (*n* = 3). Different letters indicate a significant difference at *P* < 0.05.

To reveal the effect of *PtCAT2* overexpression on H_2_O_2_ accumulation under oxidative stress, we incubated the leaves of WT and transgenic *Arabidopsis* plants in different concentrations of H_2_O_2_ solution. When exposed to either 0.5% or 1.0% H_2_O_2_, the leaves of three transgenic lines were lusher than WT (**Figure 6A**) and had higher chlorophyll content (**Figure 6B**), indicating that *PtCAT2* overexpression enables plants to degrade H_2_O_2_ and maintain their chlorophyll content.

We also monitored the water loss rate of the WT and transgenic leaves within 5 h after their detachment from the stems. The results showed that though the water content decreased in the WT and transgenic leaves, the transgenic lines maintained higher water content than the WT (*P*<0.05). This suggested that the *PtCAT2*- transgenic plants exhibited an improved water retention capacity (**Figure 6C**).

### 3.7 PtCAT2 altered the expression levels of the endogenous genes

**Figure 7:**
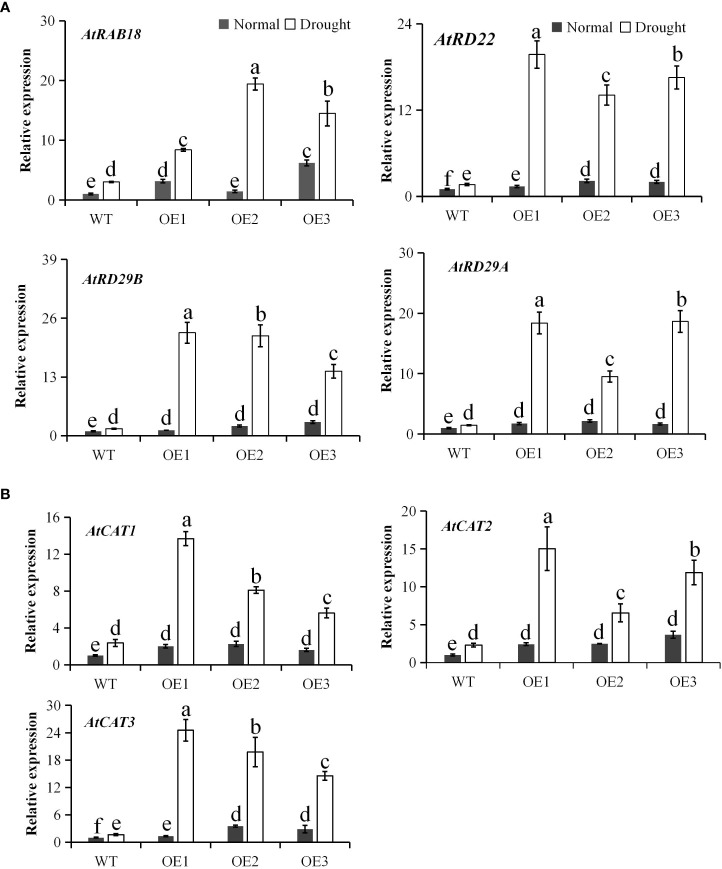
Expression of endogenous genes involved in drought stress response and CAT homologs in *Arabidopsis*. Real time RT-PCR analysis of wild-type (WT) and transgenic lines before and after 7 d of water deficit. **(A)** Drought stress related genes *AtRAB18*, *RESPONSIVE TO DEHYDRATION 22* (*AtRD22*), *RESPONSIVE TO DEHYDRATION 29B* (*AtRD29B*), *RESPONSIVE TO DEHYDRATION 29A* (*AtRD29A*). **(B)** Three endogenous *CAT* homologs (*AtCAT1*, *AtCAT2* and *AtCAT3*) in *Arabidopsis*. OE1, OE2 and OE3 represent three independent transgenic lines. Values are presented as means ± SD (*n* = 3). Different letters indicate a significant difference at *P* < 0.05.

Since *PtCAT2* overexpression enhanced drought tolerance in transgenic *Arabidopsis* plants, expression patterns of the drought stress-related genes and endogenous *CATs* were quantified in the WT and transgenic *Arabidopsis* lines under normal and drought conditions. The expression levels of drought stress-related genes, *AtRAB18, AtRD22, AtRD29B* and *AtRD29A*, were similar between WT and transgenic lines under normal conditions but were significantly upregulated in the transgenic lines compared to the WT under drought treatment (*P*<0.05) (**Figure 7A**). Moreover, the expression levels of the *Arabidopsis* endogenous *CATs* (*AtCAT1*, *AtCAT2* and *AtCAT3*) were also significantly upregulated in the transgenic lines than the WT under drought stress. In particular, *PtCAT3* had >10 folds upregulation in the three transgenic lines compared to the WT (**Figure 7B**). These results suggest that *PtCAT2* overexpression activates the expressions of drought stress-related genes and endogenous *CATs* to confer drought tolerance in plants.

## 4 Discussion

In this study, a novel class II CAT gene *PtCAT2* from *P. ternata* was functionally demonstrated to regulate plant drought tolerance. This effect was attributed to the enhanced H_2_O_2_-scavenging capacity and intact membrane integrity maintenance induced by *PtCAT2* overexpression and upregulation of the endogenous genes involved in drought stress.

Plants respond to water deficit stress by producing drought stress signals such as ROS and ABA (Yang et al., 2021). These signals lead to morphological and physiological changes, presenting as retarded growth, withering, or even plant death. Under drought stress, the balance between ROS production and clearance is disrupted (Yang et al., 2021), resulting in multiple cytological effects. One of these effects is plasma membrane lipid peroxidation (Noctor et al., 2014), which directly correlates with the integrity degree of the plasma membrane. In this study overexpression of *PtCAT2*, located at the membrane and cytoplasm, successfully enhanced the drought tolerance of *Arabidopsis*. *CAT* genes cloned from various plant species like *Arabidopsis* (Yang et al., 2019), *Ipomoea batatas* (Yong et al., 2017) and *Brassica oleracea* (Chiang et al., 2014) reportedly responded to multiple abiotic conditions. However, unlike the previously reported *CATs* involved in multiple abiotic responses (Yong et al., 2017; Zhou et al., 2017), *PtCAT2* overexpression specifically enhanced drought tolerance but not other abiotic stresses. Since mitochondria and chloroplast are the active sources of ROS (Mittler, 2017), the capacity of transgenic *Arabidopsis* to maintain the functions of these two organelles was evaluated under oxidative stress. Transgenic plants performed better oxygen consumption rate of mitochondria and chlorophyll under drought or exogenous H_2_O_2_ treatments, as well as the lower water loss rate, and membrane integrity. Our results provided insights into the biochemical mechanisms of CAT in response to drought stress, through alleviating the damage of ROS-producing organelles and maintaining membrane integrity.

The inducible expression profile of genes is usually determined by the cis-regulatory elements (Zemlyanskaya et al., 2021). An *in silico* analysis was performed to identify cis-elements in the promoter region. Known drought-responsive elements *MYCCONSENSUSAT* and *MYCATRD22*, which can bind the upstream bHLH transcription factors, were identified (Zhu et al., 2017). ABRE, the ABA-responding cis-element (Fujita et al., 2005), was also identified in the promoter region. This suggests that *PtCAT2* might respond to drought stress via an ABA-mediated signaling pathway. The *RESPONSIVE TO DEHYDRATION 22* (*RD22*) gene acts as a molecular link between ABA signaling and drought stress response (Harshavardhan et al., 2014). RABs are also known for their involvement in ABA accumulation under drought stress (Yang et al., 2012; Li et al., 2017). qRT-PCR analysis suggested that the *AtRD22* and *AtRAB18* levels were much higher in the *PtCAT2*-transgenic lines than in the WT under drought stress, suggesting that *PtCAT2* overexpression probably activates the linker between drought and ABA signaling. Moreover, the drought memory genes *Response to Desiccation* (RD) *29A* and *RD29B* (Liu et al., 2020) were also upregulated in transgenics compared with the WT. This implies that the enhanced drought tolerance in *PtCAT2*-transgenic plants was probably mediated by ABA signaling. Unlike the studies on drought tolerance regulation via stomata closure (Huber et al., 2019; Li et al., 2019), which may compromise the yield, our study provides a potentially useful approach for genetic improvement. Interestingly, the endogenous *CAT* genes (*AtCAT1*, *AtCAT2* and *AtCAT3*) were also upregulated in transgenic lines, and their induction folds were much higher than WT under drought treatment. Similar phenomenon has been observed previously (Yu et al., 1999), by ectopically expressing tobacco class II CAT gene *CatNt* to activate the endogenous homologous genes which regulate disease resistance in potatoes. This is explained by a positive and negative feedback loops within the salicylic acid signalling pathways (Yu et al., 1999). Thus, we speculate that *PtCAT2* overexpression may activate a similar signal transduction on its endogenous homologs under drought stress. The detailed mechanisms involved in this interaction necessitate further study.

## 5 Conclusion

This study functionally characterized a class II CAT gene, *PtCAT2*, from *P. ternata*, which specifically enhanced the tolerance of *Arabidopsis* plants to drought. This specific response could be attributed to its role in scavenging H_2_O_2_ to maintain the functions of ROS-producing organelles and membrane integrity, and upregulating its endogenous genes involved in drought stress responses. These results may facilitate the genetic breeding of *P. ternata* for enhanced drought tolerance.

## Data availability statement

The original contributions presented in the study are included in the article/**Supplementary Material**. Further inquiries can be directed to the corresponding authors.

## Author contributions

FLZ, YBD and JPX conceived this study. JJX, ND, TCD and HZ performed most of the experiments. JJX and TX analyzed the data. FZ performed the bioinformatics analysis. FLZ, YBD and JPX wrote the manuscript, with contribution and approval from all authors. All authors contributed to the article and approved the submitted version.
