# Supplementary material for: A novel Pinellia ternata catalase gene PtCAT2 regulates drought tolerance in Arabidopsis by modulating ROS balance
Source: Front Plant Sci. 2023 Oct 2;14:1206798. doi: 10.3389/fpls.2023.1206798 (PMC10577230; doi:10.3389/fpls.2023.1206798)
Supplement: Supplementary file 1 [file DataSheet_1.doc]

**Supplementary Files**

| Primer | Sequence | Feature |
| --- | --- | --- |
| PtCAT2-Full | CCCGGGATGGATCCCTACAAGTACCGC | Vector construction and transgenics identification |
|  | TCTAGATCACATGCTTGGTTTCATGTTGA |  |
| HYG | CTATTTCTTTGCCCTCGGAC | Transgenics identification |
|  | AAGCCTGAACTCACCGCGAC |  |
| AtTUB | TTAACACATGCAAGTCGGACG | Internal control of *Arabidopsis* for quantitative fluorescence PCR |
|  | GAGACCTCAGTAGACAAAGCACATC |  |
| PtCAT2-q | ATGAAATCCGCAGCATCTGG | Quantitative fluorescence PCR |
|  | ATGTTGAGATGACCGGCAAC |  |
| Pt18SRNA | CGCATATAAATAAACGGAGGAA | Internal control of *Pinellia ternata* for quantitative fluorescence PCR |
|  | GACGCTTCTACAGACTACA |  |
| PtCAT2-Subcellular | GAATTCATGGATCCCTACAAGTACCGC | Subcellular location vector construction |
|  | CCCGGGCATGCTTGGTTTCATGTTGA |  |
| AtRD22 | TTATTGAAGGTAGTGGCGATTG | Quantitative fluorescence PCR |
|  | ATGGAGAGTTGGGAATGG |  |
| AtRD29B | GTGAAGATGACTATCTCGGTGGTC | Quantitative fluorescence PCR |
|  | TACCAAGAGACTCAGCAATCTCTG |  |
| AtRAB18 | CATGATGACCTGGCAACTTC | Quantitative fluorescence PCR |
|  | AGCTCTAGCTCGGAGGATGA |  |
| AtRD29A | AGGAACCACCACTCAATCACA | Quantitative fluorescence PCR |
|  | GCTCATGCTCATTGCTTTGT |  |
| AtCAT1 | AGGAGCCAATCACAGCC | Quantitative fluorescence PCR |
|  | TCAAGACCAAGCGACCA |  |
| AtCAT2 | AACTCCGCCTGCTGTCTG | Quantitative fluorescence PCR |
|  | ATAGGGCATCAATCCATC |  |
| AtCAT3 | TCACAGCCACGCCACTAA | Quantitative fluorescence PCR |
|  | AGAACCAAGCGACCAACC |  |

**Table S1** Primer sequences used in this study.


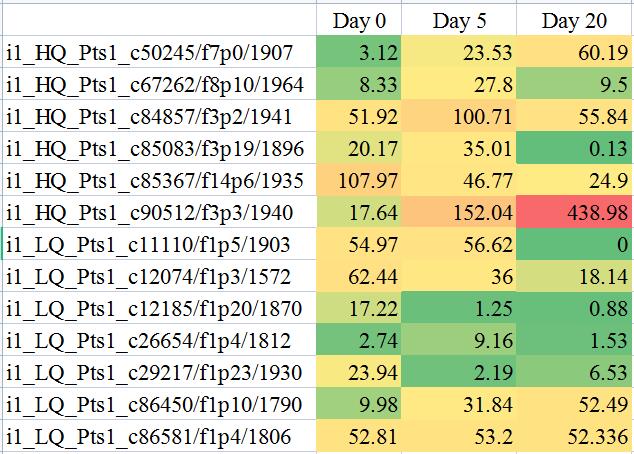


**Figure S1.** Expression profiles of candidate catalase genes identified from *Pinellia ternata* seedlings treated with water deficit for 0, 5 and 10 days. The numerical values are the FPKM of each gene. The red background indicates higher expression level while the green shows lower expression level.

**
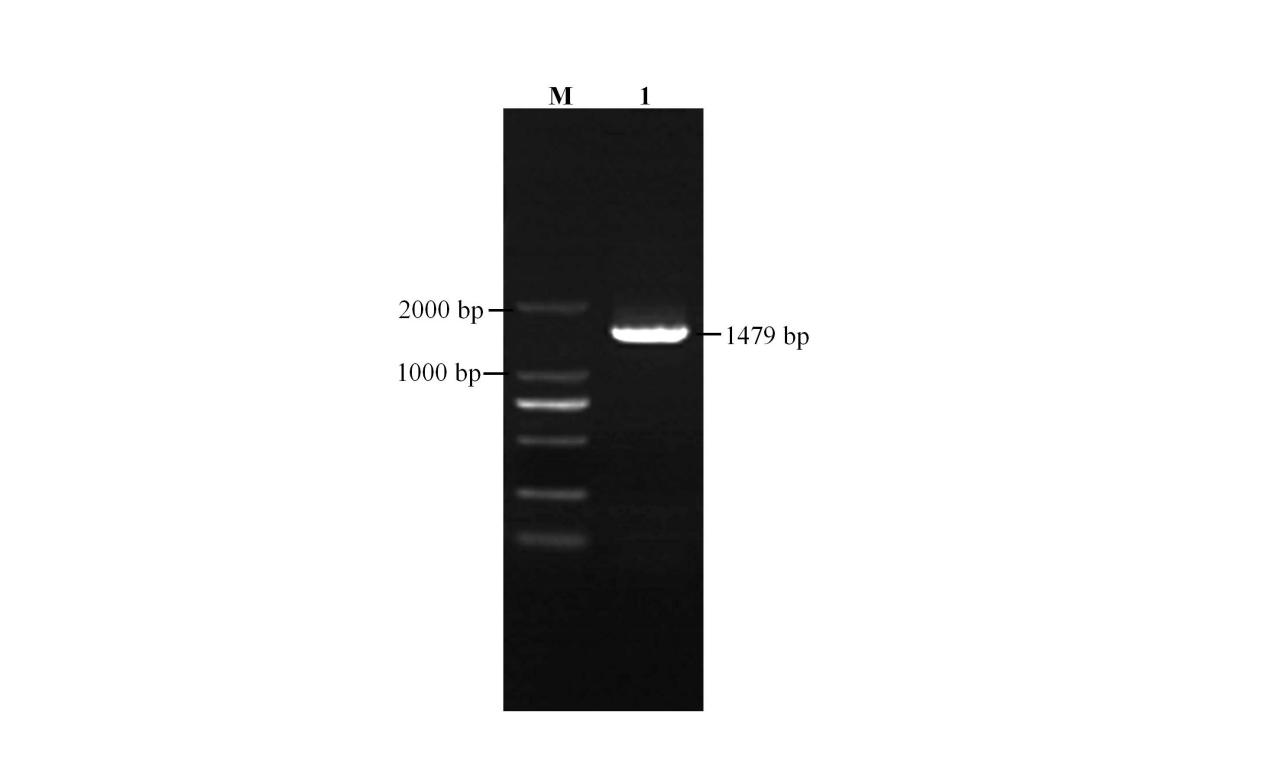
Figure S2.** Electrophoresis of PCR on *PtCAT2*. M, DL 2000 DNA marker; 1, *PtCAT2* cDNA.


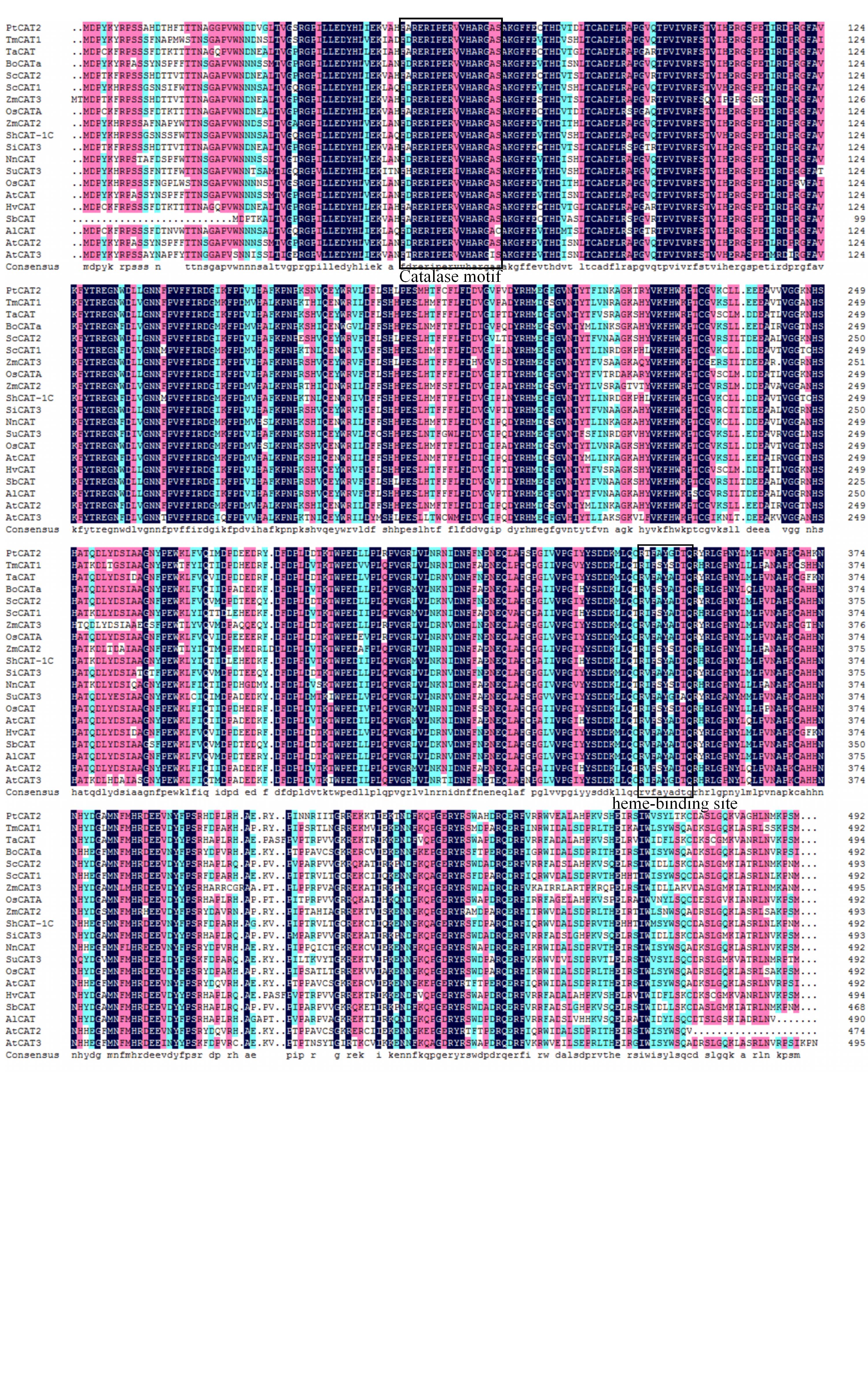
**Figure S3.** Sequence alignment of PtCAT2 with the CATs from other plants.

**
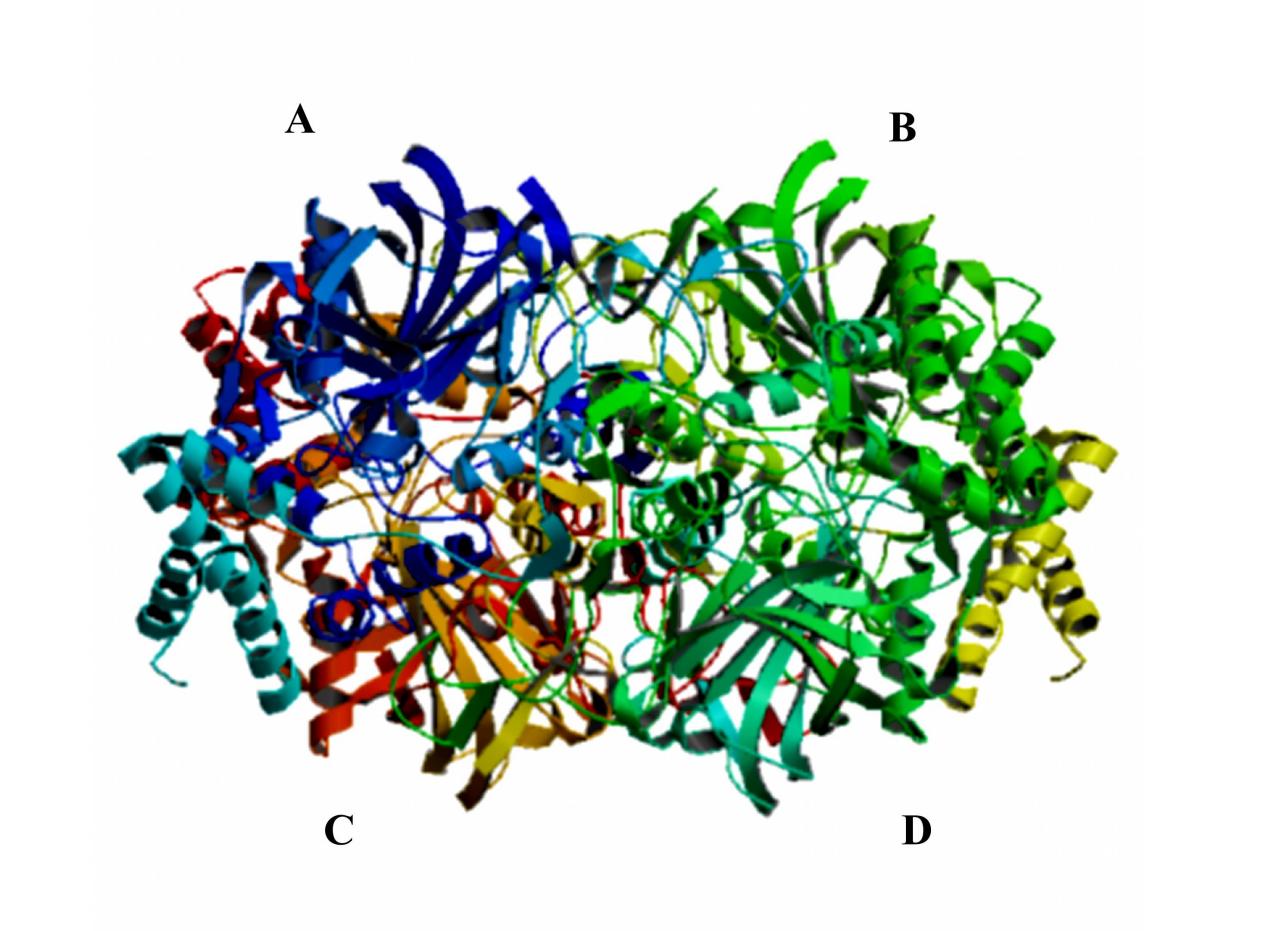
Figure S4.** Predicted 3D-structure of PtCAT2 protein. A-D indicates typical catalase domains.


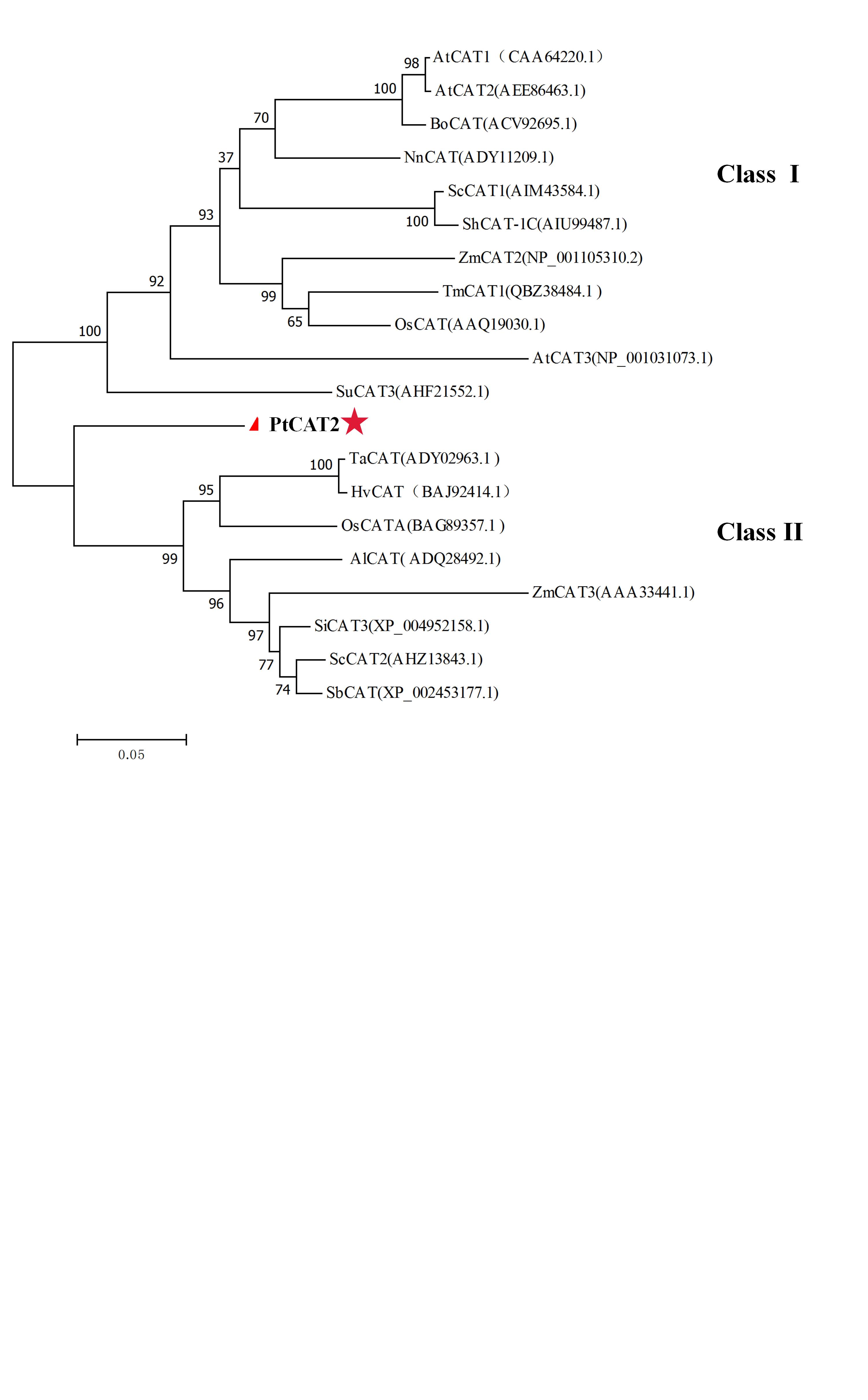


**Figure S5.** Phylogenetic analysis of PtCAT2 with functionally characterized catalases from other plant species. The phylogenetic tree was constructed using the maximum-likelihood method with 1000 bootstraps. Tm, *Triticum monococcum*; Ta, *Triticum aestivum*; Bo, *Brassica oleracea*; Sh, *Saccharum hybrid*; Zm, *Zea mays*; Os, *Oryza sativa*; Si, *Setaria italica*; Nn, *Nelumbo nucifera*; Su, *Selenicereus undatus*; At, *Arabidopsis thaliana*; Hv, *Hordeum vulgare*; Sb, *Sorghum bicolor*; Al, *Aeluropus littoralis*.


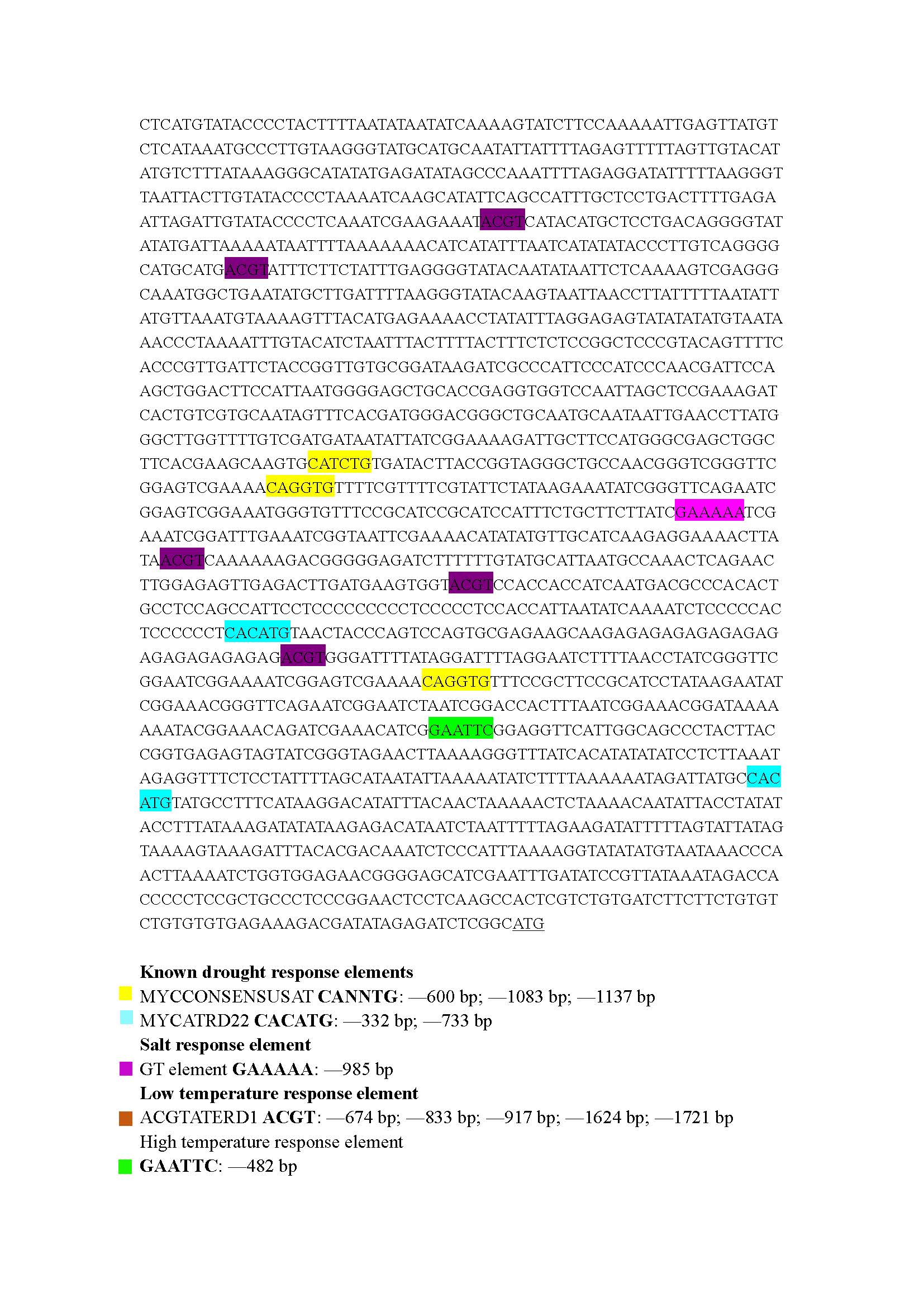
**Figure S6.** Cis elements in the promoter of *PtCAT2* predicted with PLANTCARE.

**
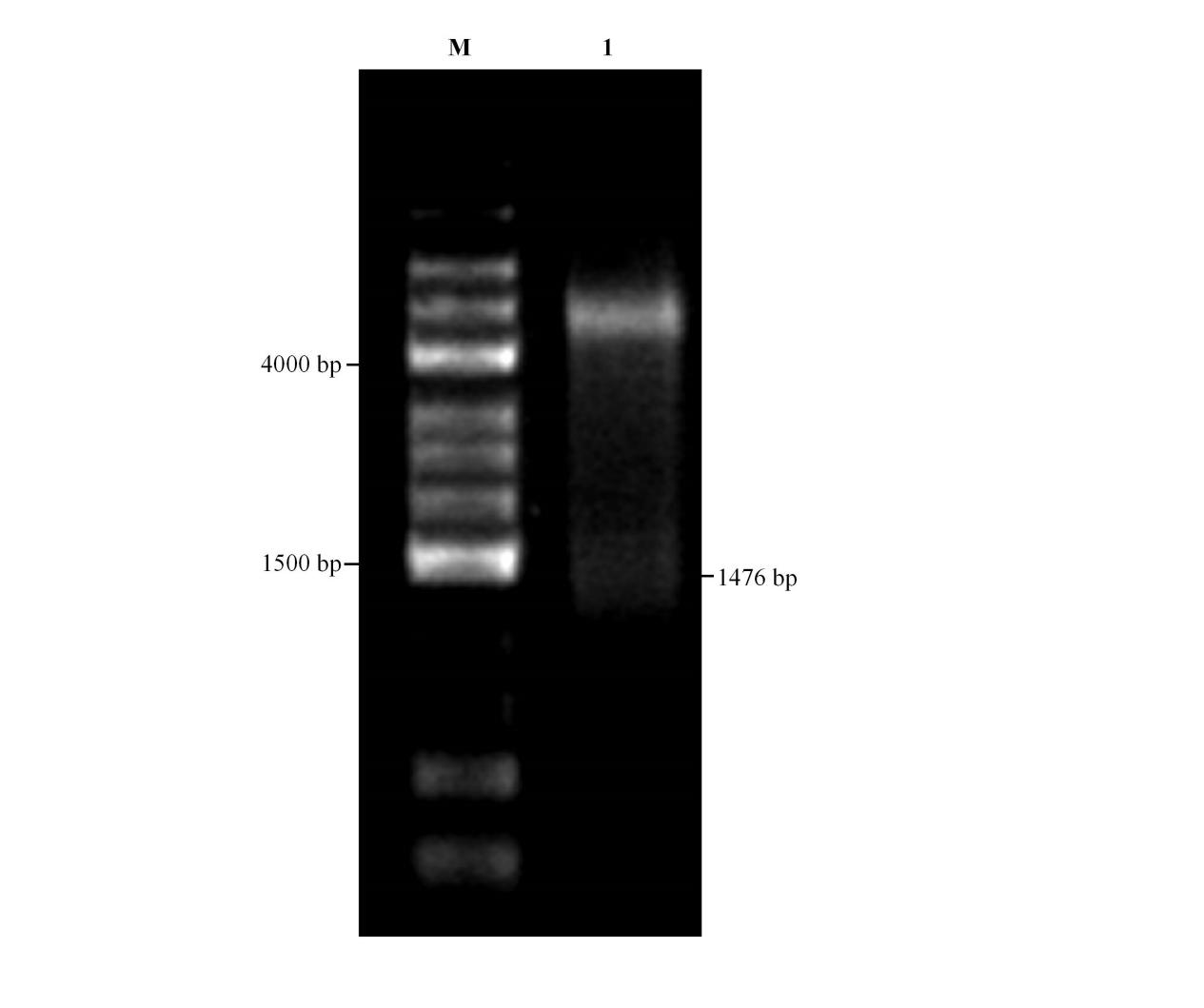
Figure S7.** Double enzyme digestion identification of subcellular localization vector for *PtCAT2.* M, 1 kb DNA marker; 1, recombinant vector.
